# Association between glaucoma severity and driving cessation in subjects with primary open-angle glaucoma

**DOI:** 10.1186/s12886-018-0788-0

**Published:** 2018-05-23

**Authors:** Aya Takahashi, Kenya Yuki, Sachiko Awano-Tanabe, Takeshi Ono, Daisuke Shiba, Kazuo Tsubota

**Affiliations:** Department of Ophthalmology, Keio University School of Medicine, Shinanomachi 35, Shinjyuku-ku, Tokyo, Japan

## Abstract

**Background:**

The aim of this study, which included a baseline cross-sectional study and a 3-year follow-up prospective study, was to investigate the association between glaucomatous visual field damage and driving cessation in subjects with primary open-angle glaucoma (POAG).

**Methods:**

A total of 211 POAG subjects divided into 3 groups according to POAG severity (mild, moderate, or severe) in the better eye were enrolled along with 148 control subjects; subjects were asked about changes in their driving status. In the 3-year follow-up study, 185 of the POAG subjects and 80 of the controls annually reported their driving status. Adjusted odds ratios and 95% confidence intervals for the prevalence and incidence of driving cessation were estimated with a multiple logistic regression model.

**Results:**

In the original cross-sectional study, 11/148 (7%) members of the control group reported having given up driving over the previous 5 years; the corresponding figures for the mild POAG, moderate POAG, and severe POAG groups were 9/173 (5%), 0/22 (0%), and 5/16 (31%), respectively (*p* = 0.001, Fisher’s exact test), with severe POAG found to be associated with driving cessation after adjustment for age, gender, systemic hypertension, and diabetes mellitus (odds ratio 11.52 [95% CI 2.87-46.35], ref. control, *p* = 0.001). In the follow-up study, the proportions of subjects who ceased driving were 1/80 (1.3%) in the control group, 8/152 (5.3%) in the mild POAG group, 5/22 (22.7%) in the moderate POAG group, and 2/11 (18.2%) in the severe POAG group (*p* = 0.001, Fisher’s exact test). Moderate POAG and severe POAG in the better eye were found to be associated with driving cessation after adjustment for age, gender, systemic hypertension, and diabetes mellitus (moderate POAG in the better eye: odds ratio 37.7 [95% CI 3.7-383.8], ref. control, *p* = 0.002, and severe POAG in the better eye: odds ratio 52.8 [95% CI 3.5-797.0], ref. control, *p* = 0.004).

**Conclusion:**

Moderate and Severe POAG in the better eye is associated with driving cessation.

## Background

Driving cessation is associated with a number of adverse outcomes, including depression [1, 2], declines in physical and social function [3], admission to long-term care [4], and mortality [5].

Glaucoma is the second leading cause of blindness in the world [6]. In glaucomatous optic neuropathy, retinal ganglion cells are slowly and progressively destroyed, with a concomitant loss of peripheral and central vision. Age is a significant risk factor for glaucoma [7], so the number of elderly drivers with glaucoma can be expected to increase in the future. Several reports have shown that subjects with glaucoma are likely to stop driving [8–10]. However, little is known about the association between glaucoma and driving cessation, and most previous studies have had cross-sectional designs. The aim of our study was to investigate the association between glaucomatous VF damage and driving cessation in subjects with POAG in both a cross-sectional and a prospective study. Our hypothesis was that severe glaucoma in the better eye is associated with driving cessation.

## Subjects and methods

The procedures followed in this study conformed to the tenets of the Declaration of Helsinki and to national (Japanese) and institutional (Keio University School of Medicine) regulations. The study was approved by the Ethics Committee of Keio University School of Medicine (#2010293). All study subjects gave informed, written consent prior to enrolment.

### Study design and subject enrolment

This study consisted of a baseline cross-sectional study and a 3-year follow-up prospective cohort study. Descriptive research design, baseline evaluation of subjects with glaucoma, diagnostic criteria for POAG, and exclusion criteria were shown in our previous paper [11, 12]. This is a sub-analysis of our two previous reports.

### Baseline question on driving status

All study subjects answered the following question (translated from the original Japanese) at the baseline ophthalmic examination:

1. Do you have a driver’s license? (Yes/No/Previously)

Subjects who answered “previously” were included in the prevalence of driving cessation data.

Demographic information recorded for all subjects included age, sex, height, weight, alcohol intake (yes/no), smoking (yes/no/previous), and current and previous illnesses (e.g., systemic hypertension, diabetes mellitus, depression, brain infarction).

### Follow-up question on driving status

All study subjects were asked the same question about whether they had a driver’s license every 12 months (± 1 month) for 3 years after they answered it at baseline. Those who answered “yes” at baseline and “previously” during follow-up were included in the incidence of driving cessation data.

### Glaucoma severity grading

For the purposes of this study, we defined mild POAG as a VF defect corresponding to a mean deviation (MD) of − 6 dB or better in the better eye, moderate POAG as corresponding to an MD of > − 6 dB to − 12 dB in the better eye, and severe POAG as an MD of > − 12 dB or worse in the better eye [13]. The eye with the better VF was defined as the eye with the higher (i.e., less negative) MD.

### Statistical analysis

The one-way ANOVA and Fisher’s exact test were used to calculate statistics for the demographic, medical, and visual-function variables between subjects among the control, mild, moderate, and severe POAG group. Age, visual acuity, and MD were analyzed with ANOVA test. Scheffé post hoc tests were also performed after one-way ANOVA. Gender, prevalence of diabetes mellitus, and prevalence of systemic hypertension were analyzed with Fisher’s exact test. Adjusted odds ratios and 95% confidence intervals for the prevalence and incidence of driving cessation were estimated with a multiple logistic regression model to examine the effects of the following (possible confounding) factors on unadjusted results (forced-entry method): age, sex, prevalence of diabetes mellitus, and prevalence of systemic hypertension. A *p*-value less than 0.05 was considered statistically significant. Decimal visual acuity was converted to LogMAR visual acuity for analysis. All analyses were performed with Stata 11.2 (Stata Co. Texas. USA) software.

## Results

### Results of the cross-sectional study

A total of 211 POAG subjects divided into 3 groups according to MD in the better eye (143 men, 68 women; age: 65.5 ± 10.7 years) and 148 control subjects (77 men, 71 women; age: 67.6 ± 11.1 years) were evaluated in this study. All subjects were Japanese, and their demographic characteristics are summarized in Table 1. There were statistically significant differences in age and sex among the 4 groups; the control group had a significantly higher average age than the POAG groups (*p* < 0.001, Scheffé post hoc test). No significant differences in BCVA in the better or worse eye were observed among the groups.

**Table 1:** Demographics and characteristics of the control and POAG groups (Baseline)

Glaucoma severity	Control	Mild glaucoma	Moderate glaucoma	Severe glaucoma	*P* value
Number	148	173	22	16	
Age (years)	72.2 ± 6.3	69.4 ± 6.1	70.4 ± 5.9	70.9 ± 6.9	0.002
Gender (male/female)	77/71 (52.0%/48.0%)	115/58 (66.5%/33.5%)	14/8 (63.6%/36.4%)	14/2 (87.5%/12.5%)	0.007
VA in the better eye (LogMar)	0.006 ± 0.03	0.003 ± 0.02	0.009 ± 0.03	0.006 ± 0.02	0.93
VA in the worse eye (LogMar)	0.02 ± 0.05	0.02 ± 0.04	0.02 ± 0.05	0.02 ± 0.05	0.95
MD in the better eye (dB)	–	−1.5 ± 1.7 [+ 2.2--6.0]	−8.2 ± 1.4 [− 6.2--11.9]	−18.2 ± 5.1 [− 12.6--29.0]	0.0001
MD in the worse eye (dB)	–	−6.3 ± 5.9 [+ 0.24--31.0]	− 14.7 ± 5.2 [− 6.2--27.4]	−21.6 ± 5.0 [− 12.7--30.4]	0.0001
Diabetes mellitus (Yes)	16/148 (10.8%)	32/173 (18.5%)	4/22 (18.2%)	3/16 (18.8%)	0.27
Systemic hypertension (Yes)	68/148 (45.9%)	65/173 (37.6%)	6/22 (27.3%)	4/16 (25.0%)	0.13

Glaucoma severity was determined on the basis of visual field in the better eye

Mean ± standard deviation [range]. Age, visual acuity, and MD were analyzed with ANOVA test. Gender, prevalence of diabetes mellitus, and prevalence of systemic hypertension with Fisher’s exact test

*Abbreviations*: *POAG* primary open-angle glaucoma, *VA* visual acuity, *MD* mean deviation, *dB* decibel

**Fig. 1:**
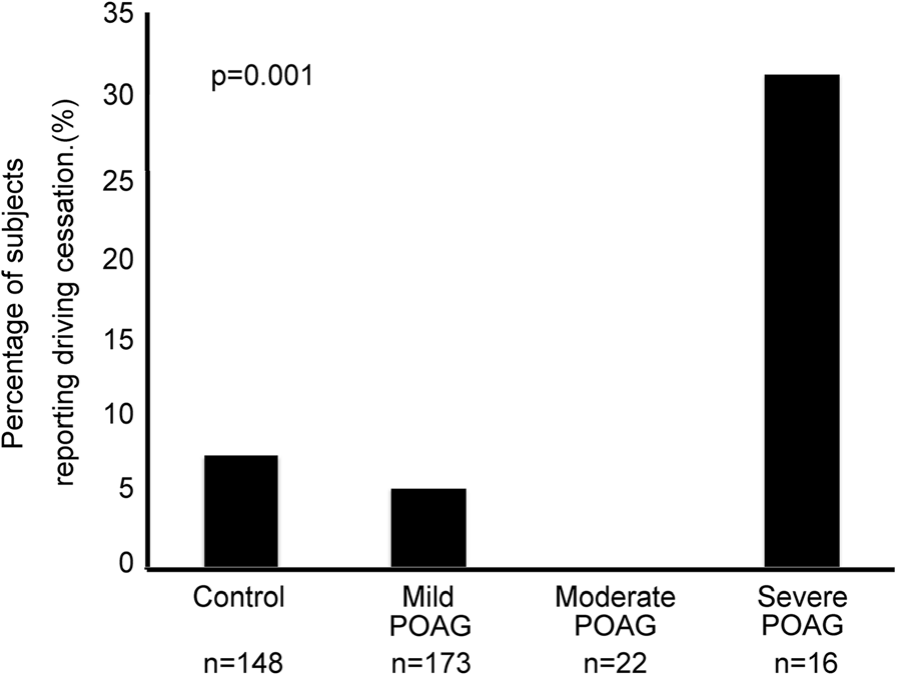
Percentage of subjects found to have given up driving in the baseline cross-sectional study. The proportions and percentages were 11/148 (7%) in the control group, 9/173 (5%) in the mild POAG group, 0/22 (0%) in the moderate POAG group, and 5/16 (31%) in the severe POAG group (*p* = 0.001, Fisher’s exact test). We defined mild POAG as a visual field defect corresponding to an MD of − 6 dB or better in the better eye, moderate POAG as an MD of > − 6 dB to − 12 dB in the better eye, and severe POAG as an MD of > − 12 dB or worse in the better eye

The proportion of subjects who had ceased driving in the control group was 11/148 (7%); the corresponding figures for the mild POAG, moderate POAG, and severe POAG groups were 9/173 (5%), 0/22 (0%), and 5/16 (31%), respectively (*p* = 0.001, Fisher’s exact test) (Fig. 1).

The association between POAG severity and driving cessation was analyzed with a logistic regression model. Severe POAG in the better eye (odds ratio 11.52 [95% CI 2.87-46.35], ref. control, *p* = 0.001) is associated with driving cessation after adjustment for age, gender, systemic hypertension and diabetes mellitus. Socio-demographic factors associated with driving cessation were age (odds ratio 1.16 [95% CI 1.08-1.23] years, *p* = 0.001) and female gender (odds ratio 2.83 [95% CI 1.12-7.18], ref. male gender, *p* = 0.028).

### Results of the prospective study

Of the 211 POAG subjects and 129 controls who agreed to participate in the follow-up study (19 control subjects declined), 185 POAG subjects (26/211 [12.3%] were lost to follow-up) and 80 controls (49/129 [38.0%] were lost to follow-up) answered questions annually over a period of 3 years about the possession of a driver’s license. The POAG subjects were divided into 3 groups according to their MD value in the better eye. Their demographic characteristics are summarized in Table 2. Statistically significant differences in age and prevalence of systemic hypertension were observed among the 4 groups. The control group was significantly older than the POAG groups (*p* < 0.001, Scheffé post hoc test). There were no significant differences in BCVA in the better or worse eye.

**Table 2:** Demographics and characteristics of the control and POAG groups (Follow-up)

Glaucoma severity^a^	Control	Mild glaucoma	Moderate glaucoma	Severe glaucoma	*P* value
Number	80	152	22	11	
Percentage of subjects reporting driving cessation	1 (1.3%)	8 (5.3%)	5 (22.7%)	2 (18.2%)	0.001
Age (years)	72.6 ± 6.0	69.0 ± 5.9	70.5 ± 5.9	69.5 ± 4.9	0.0002
Gender (male/female)	46/34 (57.5%/42.5%)	101/51 (66.4%/33.6%)	14/8 (63.6%/36.4%)	9/2 (81.8%/18.2%)	0.36
VA in the better eye (LogMar)	0.005 ± 0.03	0.003 ± 0.02	0.009 ± 0.03	0.009 ± 0.03	0.59
VA in the worse eye (LogMar)	0.02 ± 0.04	0.02 ± 0.04	0.02 ± 0.05	0.03 ± 0.05	0.62
MD in the better eye (dB)	–	− 1.5 ± 1.8 [+ 2.2 - -6.0]	− 8.2 ± 1.4 [− 6.2 - -11.9]	−16.9 ± 5.1 [− 12.6 - -29.0]	0.0001
MD in the worse eye (dB)	–	− 6.1 ± 5.5 [+ 0.24 - -26.8]	−14.7 ± 5.2 [− 6.2 - -27.4]	−20.4 ± 4.8 [− 12.7 - -29.0]	0.0001
Diabetes mellitus (Yes)	6/80 (7.5%)	29/152 (19.1%)	4/22 (18.2%)	2/11 (18.2%)	0.09
Systemic hypertension (Yes)	42/80 (52.5%)	57/152 (37.5%)	6/22 (27.3%)	2/11 (18.2%)	0.03

Mean ± standard deviation [range]. Age, visual acuity, and MD were analyzed with ANOVA test. Gender, prevalence of diabetes mellitus, and prevalence of systemic hypertension with Fisher’s exact test

*Abbreviations*: *POAG* primary open-angle glaucoma, *VA* visual acuity, *MD* mean deviation, *dB* decibel

^a^Glaucoma severity was determined on the basis of visual field in the better eye

**Fig. 2:**
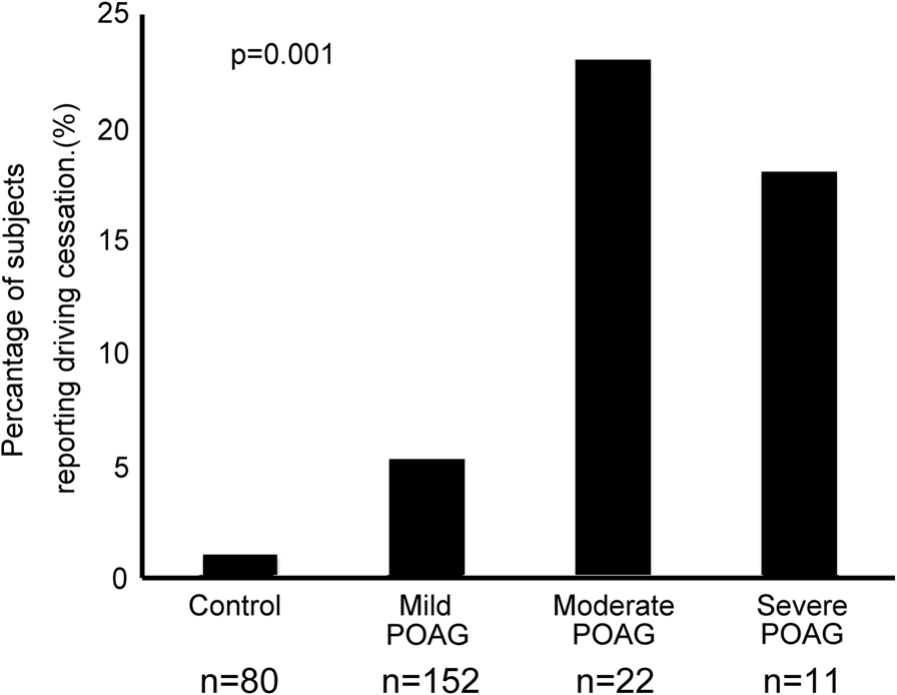
Percentage of subjects found to have given up driving in the 3-year follow-up prospective study. The proportions and percentages were 1/80 (1.3%) in the control group, 8/152 (5.3%) in the mild POAG group, and 5/22 (22.7%) in the moderate POAG group, and 2/11 (18.2%) in the severe POAG group (*p* = 0.001, Fisher’s exact test). We defined mild POAG as a visual field defect corresponding to an MD of − 6 dB or better in the better eye, moderate POAG as corresponding to an MD of > − 6 dB to − 12 dB in the better eye, and severe POAG as an MD of > − 12 dB or worse in the better eye

Among these 185 subjects and 80 controls, 16 of the subjects ceased driving in the 3-year follow-up, or 1.8% per year: 4/265 (1.5%) in the first year, 2/265 (0.8%) in the second, and 10/265 (3.8%) in the third. The proportions of subjects who ceased driving were 1/80 (1.3%) in the control group, 8/152 (5.3%) in the mild POAG group, 5/22 (22.7%) in the moderate POAG group, and 2/11 (18.2%) in the severe POAG group (*p* = 0.001, Fisher’s exact test; Fig. 2).

Significant predictor of driving cessation in this prospective study was moderate POAG in the better eye: odds ratio 37.7 [95% CI 3.7-383.8], ref. control, *p* = 0.002, and severe POAG in the better eye: odds ratio 52.8 [95% CI 3.5-797.0], ref. control, *p* = 0.004) after adjustment for age, gender, for systemic hypertension, and for diabetes mellitus. Socio-demographic factors associated with driving cessation were age (odds ratio 1.17 [95% CI 1.06-1.28] years, *p* = 0.001) and female gender (odds ratio 11.99 [95% CI 2.99-48.13], ref. male gender, *p* = 0.001).

## Discussion

This study shows that moderate and severe glaucoma in the better visual field eye is associated with driving cessation.

In the Blue Mountain Eye Study, the subjects with POAG were found to be 2.2 times more likely to stop driving (95% CI 1.3-3.9) than the controls [10]. In the Salisbury Eye Study, among the 1135 drivers (including 70 with unilateral glaucoma and 68 with bilateral glaucoma), multivariable regression analysis showed that the subjects with bilateral glaucoma were more likely to have given up driving (odds ratio 2.6, *p* = 0.002 vs. odds ratio 1.5, *p* = 0.3) than the subjects without glaucoma [8]. Van Landingham et al. also reported a higher probability of driving cessation among the glaucoma subjects than among the controls (odds ratio 4.0 [95% CI 1.1-14.7], *p* = 0.03) after multivariable adjustment. In this study, the odds of driving cessation doubled with each 5-dB decrement in MD in the better-eye (odds ratio 2.0 [95% CI 1.4-2.9], *p* < 0.001) [9]. As with our study, these previous studies suggest that severe glaucoma is associated with driving cessation. It is possible that subjects with severe glaucoma in the better eye find driving more difficult than those with unilateral glaucoma. Therefore, subjects with severe glaucoma in the better eye may be more likely to cease driving.

Ours is the first study to show an association between glaucoma severity and driving cessation in a Japanese population: previous studies showing such an association were carried out in Western countries [8–10]. In Japan, the visual standard for driver’s licensing is 0.7 (0.15 LogMar) or greater with both eye, and 0.3 or greater (0.5 LogMar) in each eye. In most of Western countries, the visual standard for driver’s licensing is 20/40 or greater with both eyes. (http://lowvision.preventblindness.org/daily-living-2/state-vision-screening-and-standards-for-license-to-drive/). Japan’s standard for vision is more restrict than western countries. Therefore, severe glaucoma subjects were more likely to fail to renew driver’s license in Japan.

Our study also showed that age and female gender were associated with driving cessation, and the Salisbury Eye Study revealed that age and female gender were risk factors for not driving [8]. The Blue Mountain Eye Study showed increasing odds of driving cessation with each decade increase in age (odds ratio 2.4 [95% CI 2.1-2.7]). Female gender was also associated with driving cessation (odds ratio 3.2 [95% CI 2.5-3.9]) in the Blue Mountain eye study [10]. These results are compatible with ours.

We acknowledge several limitations in our study. First, the main outcome relied on self-reported receipt of a driver’s license, so the results could have been affected by recall bias. Second, the number of subjects who reported driving cessation was relatively small. However, an association between worsened VF in the better eye and driving cessation was shown in both the baseline cross-sectional study and the follow-up prospective study, which suggests that the results are robust. A third weakness is that data on other possible risk factors for driving cessation, including depressive symptoms, marital status, place of residence, and economic status, were not collected, which could have led to some bias in our analysis. Fourth, it is not possible to determine if POAG was the cause of driving cessation in this study. Fifth, we acknowledge that there are intermediate steps between the first signs of functional loss and driving cessation: since severe glaucoma is associated with depressive symptoms [14], functional loss could indirectly lead to driving cessation by causing depressive symptoms. Sixth, we classified driving cessation on the basis of giving up a driver’s license, but we recognize that in fact, many older adults who stop driving voluntarily continue to hold a license; account of this should be taken in future studies. Seventh, the 95% confidence interval presented in the result section is very wide, this may be because the number of subjects who gave up driving is relatively small.

## Conclusion

Moderate and Severe glaucoma in the eye with the better visual field is associated with driving cessation, and may be a clinical predictor of driving cessation.
